# Supplementary material for: An interpretative phenomenological study about maternal perceptions of cesarean birth
Source: PLoS One. 2025 Feb 6;20(2):e0318525. doi: 10.1371/journal.pone.0318525 (PMC11801696; doi:10.1371/journal.pone.0318525)
Supplement: Attachment — Submitted filename: PONE-D-24-38372.pdf [file pone.0318525.s002.pdf]

PLOS ONE

An Interpretative Phenomenological Study about Maternal Perceptions of Cesarean Birth

--Manuscript Draft--

**Manuscript Number:**	PONE-D-24-38372
**Article Type:**	Research Article
**Full Title:**	An Interpretative Phenomenological Study about Maternal Perceptions of Cesarean Birth
**Short Title:**	Perception of CS Birth
**Corresponding Author:**	Zaid Al-Hamdan<br>Jordan University of Science and Technology<br>Irbid, Amman JORDAN
**Keywords:**	Maternal perception, mobile clinic, mode of delivery, Jordan, phenomenology, qualitative study.
**Abstract:**	Aim: To explore the perceptions of Jordanian primipara and para 1 women regarding Cesarean section delivery and provide insights for clinical practice, policy development, and future research to optimize maternity care practices and outcomes in Jordan and similar contexts.<br>Background: Global Cesarean section (CS) rates have risen steadily over recent decades. Jordan has witnessed a significant increase in CS rates. This trend has sparked interest in understanding the underlying factors behind this phenomenon and the perceptions surrounding CS delivery. Understanding these factors is essential for informing healthcare practices and promoting optimal maternal and neonatal health outcomes.<br>Methods: Forty-one primipara and para 1 women were interviewed through in-depth semi-structured interviews within seven focus groups. The analysis was conducted using interpretative phenomenological analysis with the assistance of the NVivo 12 software program.<br>Results: Three main themes were identified: The Foundation of Women's Knowledge and Cognitive Structures; The Influential Parties in Shaping Women’s Perceptions; and Transforming Perception into Action.<br>Discussion: To offer empathetic, comprehensive, and patient-centered care, healthcare professionals should discuss delivery options with pregnant mothers and factor in maternal preferences and perceptions. This study underscores the importance of supportive dialogue and informed decision-making in maternity care, advocating for a more individualized approach to childbirth.
**Order of Authors:**	Hala Bawadi
	Zaid Al-Hamdan
	Nagham Abu Shaqra
	Amer Gharaibeh
	Hasan Rawashdeh
	Asma Basha
	Majida Jallad
	. Sawsan Majali
	Heba AboShindi
	Mahmoud T aym
**Additional Information:**	
**Question**	**Response**
**Financial Disclosure**	The author(s) received no specific funding for this work.

Powered by Editorial Manager® and ProduXion Manager® from Aries Systems Corporation

Enter a financial disclosure statement that describes the sources of funding for the work included in this submission. Review the submission guidelines for detailed requirements. View published research articles from *PLOS ONE* for specific examples.<br><br>This statement is required for submission and **will appear in the published article** if the submission is accepted. Please make sure it is accurate.<br><br>**Funded studies**<br>Enter a statement with the following details:<br>• Initials of the authors who received each award<br>• Grant numbers awarded to each author<br>• The full name of each funder<br>• URL of each funder website<br>• Did the sponsors or funders play any role in the study design, data collection and analysis, decision to publish, or preparation of the manuscript?<br><br>Did you receive funding for this work?	
**Competing Interests**<br><br>Use the instructions below to enter a competing interest statement for this submission. On behalf of all authors, disclose any competing interests that could be perceived to bias this work—acknowledging all financial support and any other relevant financial or non-financial competing interests.<br><br>This statement is required for submission and **will appear in the published article** if the submission is accepted. Please make sure it is accurate and that any funding sources listed in your Funding Information later in the submission form are also declared in your Financial Disclosure statement.	no competing interests exist.

View published research articles from *PLOS ONE* for specific examples.<br><br>**NO authors have competing interests**<br><br>Enter: *The authors have declared that no competing interests exist.*<br><br>**Authors with competing interests**<br><br>Enter competing interest details beginning with this statement:<br><br>*I have read the journal's policy and the authors of this manuscript have the following competing interests: [insert competing interests here]*<br><br>* typeset	
**Ethics Statement**<br><br>Enter an ethics statement for this submission. This statement is required if the study involved:<br><br>• Human participants<br>• Human specimens or tissue<br>• Vertebrate animals or cephalopods<br>• Vertebrate embryos or tissues<br>• Field research<br><br>Write "N/A" if the submission does not require an ethics statement.<br><br>General guidance is provided below. Consult the submission guidelines for detailed instructions. **Make sure that all information entered here is included in the Methods section of the manuscript.**	Jordan University IRB

**Format for specific study types**<br><br>**Human Subject Research (involving human participants and/or tissue)**<br>• Give the name of the institutional review board or ethics committee that approved the study<br>• Include the approval number and/or a statement indicating approval of this research<br>• Indicate the form of consent obtained (written/oral) or the reason that consent was not obtained (e.g. the data were analyzed anonymously)<br><br>**Animal Research (involving vertebrate animals, embryos or tissues)**<br>• Provide the name of the Institutional Animal Care and Use Committee (IACUC) or other relevant ethics board that reviewed the study protocol, and indicate whether they approved this research or granted a formal waiver of ethical approval<br>• Include an approval number if one was obtained<br>• If the study involved *non-human primates*, add *additional details* about animal welfare and steps taken to ameliorate suffering<br>• If anesthesia, euthanasia, or any kind of animal sacrifice is part of the study, include briefly which substances and/or methods were applied<br><br>**Field Research**<br><br>Include the following details if this study involves the collection of plant, animal, or other materials from a natural setting:<br>• Field permit number<br>• Name of the institution or relevant body that granted permission	
**Data Availability**<br><br>Authors are required to make all data underlying the findings described fully available, without restriction, and from the time of publication. PLOS allows rare exceptions to address legal and ethical concerns. See the PLOS Data Policy and FAQ for detailed information.	Yes - all data are fully available without restriction

Powered by Editorial Manager® and ProduXion Manager® from Aries Systems Corporation

A Data Availability Statement describing where the data can be found is required at submission. Your answers to this question constitute the Data Availability Statement and **will be published in the article**, if accepted.<br><br>**Important:** Stating 'data available on request from the author' is not sufficient. If your data are only available upon request, select 'No' for the first question and explain your exceptional situation in the text box.<br><br>Do the authors confirm that all data underlying the findings described in their manuscript are fully available without restriction?	
**Describe where the data may be found in full sentences. If you are copying our sample text, replace any instances of XXX with the appropriate details.**<br><br>• If the data are **held or will be held in a public repository**, include URLs, accession numbers or DOIs. If this information will only be available after acceptance, indicate this by ticking the box below. For example: *All XXX files are available from the XXX database (accession number(s) XXX, XXX.).*<br>• If the data are all contained **within the manuscript and/or Supporting Information files**, enter the following: *All relevant data are within the manuscript and its Supporting Information files.*<br>• If neither of these applies but you are able to provide **details of access elsewhere**, with or without limitations, please do so. For example:<br><br>*Data cannot be shared publicly because of [XXX]. Data are available from the XXX Institutional Data Access / Ethics Committee (contact via XXX) for researchers who meet the criteria for access to confidential data.*<br><br>*The data underlying the results presented in the study are available from (include the name of the third party*	All relevant data are within the manuscript and its Supporting Information files.

and contact information or URL).<br>• This text is appropriate if the data are owned by a third party and authors do not have permission to share the data.<br><br>* typeset	
Additional data availability information:	

Manuscript Click here to access/download;Manuscript;Main documnet.docx

An Interpretative Phenomenological Study about Maternal Perceptions of Cesarean Birth

1. Dr. Hala Bawadi Professor, Department of Maternal and Child Health, School of Nursing, The University of Jordan h.bawadi@ju.edu.jo
2. Dr. Zaid Al-Hamdan Professor. Community and Mental health department, Faculty of Nursing , Jordan University of Science and Technology zaid_hamdan@hotmail.com; zmhamdan@just.edu.jo*
3. Dr. Nagham Abu Shaqra, MD, Senior Population and Health Advisor/Project Management Specialist/ Office of Population and Family Health | USAID Jordan nabushaqra@usaid.gov
4. Dr. Amer Gharaibeh, the chief of obs/gyn department at royal medical services and JMMSR NAG member (agharaibeh@hotmail.com)
5. Dr. Hasan Rawashdeh Associate Professor at Jordan University of Science and Technology **hmrawashdeh@just.edu.jo**
6. Asma Basha, Prof Director of Obstetrics and gynecology, The University of Jordan and Jordan University Hospital bashaasma@yahoo.com
7. Dr. Majida Jallad, Consultant Obstetrics and gynecology drmajallad@hotmail.com
8. Dr. Sawsan Majali, Senior Health System Stregnthening Advisor, at USAID Health Services Quality accelerator Activity/ university Research Company (smajali@ic.urc-chs.com)
9. Heba AboShindi Surveillance, Data Management and MMSR Advisor,USAID Health Services Quality Accelerator Activity habushindi@urc-chs.com
10. Dr. Mahmoud Taym, Clinical Quality Improvement Specialist/ Obs/gyn specialist at USAID Health Services Quality accelerator Activity/ university Research Company (mtayem@URC-CHS.COM)

*Corresponding author

Abstract

Aim: To explore the perceptions of Jordanian primipara and para 1 women regarding Cesarean section delivery and provide insights for clinical practice, policy development, and future research to optimize maternity care practices and outcomes in Jordan and similar contexts.

Background: Global Cesarean section (CS) rates have risen steadily over recent decades. Jordan has witnessed a significant increase in CS rates. This trend has sparked interest in understanding the underlying factors behind this phenomenon and the perceptions surrounding CS delivery. Understanding these factors is essential for informing healthcare practices and promoting optimal maternal and neonatal health outcomes.

Methods: Forty-one primipara and para 1 women were interviewed through in-depth semi-structured interviews within seven focus groups. The analysis was conducted using interpretative phenomenological analysis with the assistance of the NVivo 12 software program.

Results: Three main themes were identified: The Foundation of Women's Knowledge and Cognitive Structures; The Influential Parties in Shaping Women's Perceptions; and Transforming Perception into Action.

Discussion: To offer empathetic, comprehensive, and patient-centered care, healthcare professionals should discuss delivery options with pregnant mothers and factor in maternal preferences and perceptions. This study underscores the importance of supportive dialogue and informed decision-making in maternity care, advocating for a more individualized approach to childbirth.

Keywords: Maternal perception, mobile clinic, mode of delivery, Jordan, phenomenology, qualitative study.

Introduction

The global rise in Cesarean Section (CS) rates has become a concern to healthcare providers, policymakers, and expectant mothers alike. The World Health Organization (WHO) states that an optimal CS rate falls between 10% and 15% of all births (1). In Jordan, recent years have seen a rate of approximately 25% (2). Data collected from different sectors of Jordan's healthcare system reported the following rates for 2015–2017: 40.4% (±2.6) in university hospitals, 39.1% (±1.8) in private hospitals, 36.1% (±0.2) in military hospitals, and 27.4% (±0.7) in governmental hospitals (3). These statistics have prompted researchers to investigate the factors contributing to the prevalence of CS deliveries and to understand the perceptions of CS among pregnant Jordanian women, specifically those who are experiencing their first birth (primipara) or have previously given birth once (para 1).

Numerous factors influence a woman's choice to undergo a CS delivery. These factors include medical indications, cultural norms, socioeconomic status, and individual preferences. Salem (2021) pointed to previous CS (33.6%), abnormal presentation (20.3%), and patient request (16%) as common indications for CS in Jordan. CS, when necessary, is undoubtedly a life-saving intervention. However, when performed without medical justification, it can put both mother and baby at risk, leading to increased rates of maternal morbidity and mortality, neonatal respiratory complications, and longer recovery times (4). **Therefore,** we need to understand how Jordanian primipara and para 1 women perceive CS delivery to promote birthing practices and achieve optimal maternal and neonatal outcomes.

Previous research on CS delivery has focused on healthcare professionals' perspectives rather than those of pregnant women. This qualitative study seeks to explore this population's perceptions, beliefs, and decision-making processes surrounding CS. This will enable healthcare providers to tailor care to serve expectant mothers better, ultimately promoting evidence-based, woman-centered maternity care in Jordan.

This qualitative study investigated the perceptions of Jordanian primipara and para 1 women regarding CS delivery. In-depth interviews were conducted to reveal participants' attitudes and knowledge about CS, its risks and benefits, and their reasons for opting for or rejecting this mode of delivery. The findings serve to inform clinical practice, policy development, and future research endeavors to enhance maternity care practices and outcomes in Jordan and similar contexts.

Methods

Study design

An interpretative phenomenological approach was adopted because it allows participants to respond to in-depth, probing questions, express their opinions, and expand upon their experiences (5).

Setting and participants

This Jordanian study recruited its participants from two public hospitals and two health centers. Three focus groups were held with primiparous women (2nd and third trimesters) at hospital antenatal care (ANC) clinics. Four other focus groups were conducted with para 1 women (14-50 days postpartum) who brought their infants to the centers for vaccination and neonatal

screening. Purposive sampling was employed, utilizing a maximum variation strategy (6) Based on age, education level, and family income. In total, 41 women who met the criteria and agreed to participate were divided into seven groups of 5-7, The sample size was determined based on theme saturation (7).

Data collection and procedures

Focus groups and in-depth semi-structured interviews were used for data collection as they are effective approaches for exploring perceptions and lived experiences (8). Participants expressed themselves freely, and the researchers posed questions uncovering in-depth data. To ensure comprehensive coverage of relevant topics (9). The research team developed an interview guide. The lead author conducted the interviews, each lasting 60 - 70 minutes. The participants' recruitment started after IRB was granted on Feb, 1 to March 30, 2024.

Data analysis

Upon completion of each interview, the recorded data underwent a verbatim transcription to Arabic and then was translated into English. For data organization and categorization, the English transcripts were entered into the NVivo 12 software (QSR International Pty Ltd, Doncaster, Victoria, Australia), which labels each paragraph with phrases representing specific ideas. The first author analyzed data using the interpretative phenomenological analysis framework (10). The NVivo-generated categorized data was manually assigned as single phrases to groups of linked sub-phrases, which were then listed under a significant overarching idea. The research team held comprehensive discussions to identify themes predominantly zeroing in on extracted quotes from the interviews, which were then entered into the final thematic list.

Ethical consideration

Ethical clearance was obtained from the Institutional Review Boards of the Ministry of Health and the Royal Medical Services. The women were told that participation was voluntary and that their identities would not be disclosed. Informed consent was obtained from all participants before commencing the interviews.

Trustworthiness of the study

The investigation adhered to the criteria of dependability, credibility, confirmability, and transferability to ensure its rigor (11). The first author discussed the emerging themes and their interpretations with the research team, taking their feedback into account. Some participants requested and were permitted to review the final list of themes to ensure their opinions, experiences, and needs were accurately represented. The list of the themes was sent via WhatsApp.

Results

Forty-one women were interviewed within seven focus groups. The women fell into the 19-49 age range. 17 women were primipara, and 24 were para 1. 16 women were employed, and

25 were housewives. All the husbands were employed. 19 received ANC in public health facilities, 10 in private, and 12 mixed between public and private.

It should be noted that the perceptions of our participants are not representative of all primipara and para 1 Jordanian women, whose experiences may differ. The analysis presented in this study emerged from the experiences of the women in our sample, and there were varying opinions regarding CS, with some in favor and some against. We found that women generally followed a decision-making process regarding CS based on their individual knowledge and thinking structure, each having certain factors influencing perception formation. It is important to recognize that specific contexts influence these perspectives, and the viewpoints of this subset of Jordanian primiparous and para 1 women may not be universally applicable.

The thematic analysis of the Jordanian women's perceptions regarding CS is shown in Table 1.

Table (1): Themes and sub-themes of the Jordanian women's perceptions regarding CS

THEME	SUBTHEME
**The Foundation of Women's Knowledge and Cognitive Structures**	▪ Perceived Meaning of CS<br>▪ Perceived Benefits<br>▪ Perceived Concerns about CS<br>▪ Previous Experiences
**The Parties Influential in Shaping Women's Perceptions**	▪ Trust in the Doctor<br>▪ Influence of the Husband and Gender Equity<br>▪ Role of Culture<br>▪ Maternal Health Condition

**Transforming Perception into Action**	▪ Decision on Mode of Delivery<br>▪ Recommendations for Future Mothers

The Foundation of Women's Knowledge and Cognitive Structures

This theme explains how the woman's view of CS is formed and crystallized based on the knowledge she possesses and, ultimately, her feelings regarding her preferred mode of delivery. This theme consists of several sub-themes, as follows:

Perceived Meaning of CS

Some participants concurred that a CS does not represent the typical course of labor. Conversely, a 'normal' vaginal delivery (NVD) indicates an absence of complications and a smooth progression of labor. One participant explained:

"NVD means a natural stage that the woman goes through at the end of her pregnancy. CS means there is a problem. Risk for the mother or the baby leads to having CS." (Para 1, FG4)

Some women reported that a CS represented a bearable pain compared to the intense pain of NVD.

"NVD means unbearable pain for me. Cesarean section is painful, but with strong painkillers, the pain is not comparable to the pain of labor." (Para 1, FG 1)

Perceived Benefits of CS

Some participants said that they opted for CS because it meant that they would not be exposed to suffering and pain before seeing the baby. The pain after the CS could be tolerated as it would be alleviated by the presence of the newborn:

"I prefer a CS because I will not suffer before I see my babies. I want to go to sleep and wake up to find them next to me. I feel that looking at them will ease my pain." (Primipara, FG 3)

One participant added:

"CS is easier and better for me. A CS is more comfortable, as the lady said. You sleep and wake up to find the baby next to you." (Para 1, FG1)

Some women view CS as the preferable mode of birth because it does not entail waiting for the unknown. They appreciate the scheduled nature of the procedure, allowing for clear expectations regarding the date and mode of delivery. As one participant articulated:

"I gave birth to CS. For sure, you are in pain after giving birth, but it is enough for you to enter the hospital without pain and go directly to the theatre; then, you and the baby will come out safely. You will suffer after giving birth, but God will make it easy." (Para 1, FG2)

The factor of close and continuous monitoring of the mother during childbirth was a top priority among primipara women. They explained that one of the benefits of CS is that the doctor remains present from the beginning of the operation to its end, next to the woman giving birth. While in NVD, the doctor will only be present intermittently, coming at different times to examine her:

"I intend to have a CS. The doctor is by your side. The anaesthesiologists are taking care of you and are paying attention. In NVD, it is normal for them to put you on the bed and forget about you. There is no care like that, I hear." (Primipara, FG3).

Some women also felt that a woman who delivers by CS is given extra care because she has been through surgery and needs care until the wound heals.

"A cesarean section means pampering and attention. You will stay asleep and comfortable... Oh, you will relax and be pampered for the first period." (Primipara, FG2)

Some of the women confirmed their fears that NVD would alter the shape of the vulva and size of the vagina in a way that is unattractive, and this may affect the marital relationship, even leading to possible infidelity:

"I know that the shape and size of the area will be changed after NVD. I heard the sight will be disgusting, and your husband may become disgusted to sleep with you, and he may cheat on you." (Primipara, FG2)

This idea was reiterated and confirmed by other participants, who brought to light how their husbands directly requested a CS delivery, prompted by their concerns about the change in the shape of the vulva area and expansion of the vagina.

"My husband went to the doctor and asked him for a CS. He discussed with the doctor the deformity of the area, and he couldn't bear to see this, and the doctor agreed to perform a cesarean." (Para 1, FG4)

Perceived Concerns about CS

Some participants explained that the nature and duration of the pain were uppermost in their thoughts:

"Honestly, both are painful, but I prefer NVD to CS because it will only hurt for hours, while CS will cause pain for weeks, and I will not be able to take care of my baby." (Para 1, FG3)

One primipara added that she was afraid of infection in the CS wound and was concerned about how to care for it:

"I was afraid of infection from the operation and infection of the wound. I had no other fears." (Para 1, FG3)

On the other hand, some women were aware of the dangers of CS and some complications that the mother may be exposed to, such as wound pain and possible infection, as well as long-term complications of adhesions. As for the fetus, he would be vulnerable to lack of oxygen:

"A CS means adhesions, more bleeding, and a risk to the baby of low oxygen levels, requiring the baby to enter an incubator. CS means pain at the wound site, postpartum infections, lack of movement, and back needles." (Para 1, FG2)

Some participants were excited about the CS but worried about their post-surgery condition and the resulting pain. They expressed concerns about being able to move about and care for the baby independently without help. They also thought about the future, their body shape, and the fear of sagging in the abdominal area, as one of the participants explained:

"The thing I am most afraid of is coughing or sneezing after CS because they are very painful. Also, the first walk after the operation. Also, after I go home, when I wake up for the baby at night, I will sleep on my back; how will I get up? It will be a lot of suffering." (Primipara, FG1)

Previous Experiences

Previous experiences greatly influenced the participants' perceptions regarding CS. It was clear that participants all knew about bad experiences, whether they were her own or those of a sister or friend. The trace of the experience remains engraved in the memory, often overshadowing many successful experiences. There are many women who were induced and went on to have an NVD, but what remains in the women's collective memory are the occasions when birth began with induction and ended, for mostly medical reasons, with a CS. In these cases, the women were exposed to the pain of normal delivery, added to the pain of a CS delivery:

"When I was delivered, I lived through a week of terror, induction, and internal examination, opposite to what I expected. To be honest, I have been living in terror until now, from what I suffered." (Para 1, FG 2)

Another participant added:

"I preferred CS, to be honest; after what happened to my sister, I didn't want to give birth normally. My sister was admitted for labor, and a trainee doctor came and ruptured her membrane and the baby suffocated in her abdomen and died. I will tell them that I want a CS. I learned from the experience of my sister." (Primipara, FG 1)

Some women narrated how, at the hospital, they witnessed other women who suffered during natural childbirth. They saw that there was negligence on the part of the medical staff and a lack of respect for the woman's privacy, so they felt discouraged about NVD and favoured CS:

"From the front of the operating room, I could see a normal delivery room where a woman giving birth. She was bleeding, and no one paid attention to her. I tried to call the doctors and they said nothing was wrong. They did not come and see her. There was a lot of neglect and no care. The

patient was also exposed in front of passers-by. This is unacceptable. In a CS, there is privacy, cleanliness, and care. Within a quarter of an hour, you are finished, and everything is fine, thank God." (Para 1, FG1)

Some women told stories of relatives who were handled badly at birth and ongoingly experienced the consequences. A woman told us that when her husband was born, his shoulder was dislocated, and he had spent all his life unable to use his hand normally, so she preferred CS.

"I am afraid of NVD. My husband had a normal birth, but when he was born, he had a dislocation in his shoulder. This thing affected him, and his hand is not normal until now. This thing scares me a lot. I don't want to give birth normally." (Para 1, FG2).

The Parties Influential in Shaping Women's Perceptions

This theme discusses the parties who are influential in shaping women's opinions regarding CS delivery in both positive and negative ways.

Trust in the Doctor

It was evident that most of the participants trusted their doctor's opinion. The paternalistic concept was very clear during the participants' interviews. That is, the woman firmly believes that the doctor knows what is best for her condition and can make the appropriate decision:

"He explained it to me exactly, from the beginning. He told me that I would have complications. So, I felt that he knew about my condition, and it was better to trust him." (Primipara, FG2)

"If the doctor had tried with me, I would have given birth normally. The decision is in the doctor's hands." (Para 1, FG2)

Influence of the Husband and Gender Equity

Most women had discussed the preferred method of delivery with their husbands. Most husbands respected the doctor's opinion about the mode of delivery depending on the wife's health condition. Some husbands leave the decision to the wife, allowing her to do what is appropriate and comfortable for her:

"He said the decision is in your hands and consult the doctor. No one feels the pain except you." (Primipara, FG3)

"My husband told me to do what I wanted. He said, "The important thing is that they save you." (Primipara, FG1)

"I used to talk to my husband a lot about the subject, and he encouraged me to deliver normally." (Para 1, FG3)

Role of Culture

Cultural beliefs and norms surrounding childbirth heavily influence women's perceptions of CS. Jordanian culture sees vaginal birth as more natural or traditional. On the other hand, CS may be viewed as a safer option, entailing less suffering. Cultural attitudes towards pain, childbirth, and medical interventions can all shape how women perceive CS and influence their decision-making.

Our participants differed about whether the prevailing culture encourages NVD or CS, but ultimately, they agreed that the old generation and some of the new generation encourage NVD:

"My mother and mother-in-law encourage NVD, while my friends encourage CS." (Para 1, FG 1)

Many participants felt that most of the new generation of mothers encourages CS:

"I am Primipara, but I worked as a maternity nurse. A week ago, I swear, 3 pregnant women asked for a CS. The doctor put them in for labor induction. They said that they didn't want to, so the doctor told them to sign a paper and performed a CS for them." (Primipara, FG 1)

Several women expressed the belief that some doctors actively promote CS delivery:

"I feel that women are not very enthusiastic about NVD, and doctors encourage women to have CS because of the financial factor involved in the matter. I feel that most new mothers have had cesarean births at present. My friend was pregnant with her fourth child. She had excellent conditions for a natural birth, but they referred her to have a CS without knowing the reason." (Para 1, FG 1).

Maternal Health Condition

A woman's health status and any potential complications during pregnancy can also impact her perception of CS. Pre-existing health conditions or pregnancy-related complications may increase the risk of a difficult NVD, thus encouraging the mother to consider a planned CS as a safer option for herself and her baby.

"I had no dilatation and no contractions. The cervix was closed. At the end, the baby's pulse began to decrease because the fluids were very light, because of this factor. The only reason I chose CS was because there was a threat to the life of the fetus." (Para 1, FG 4)

"I conceived through IVF. What made me give birth by CS was that I gave birth to her in the eighth month. My water broke, and I had to go to the hospital. There, they started telling me to wait, but

I told them that I was not ready to take the risk, not even 1%. I did not agree with the NVD because I told them that I had waited all these years to see the baby. That's why I requested CS." (Para 1, FG2)

Transforming Perception into Action

Perceptions of CS delivery, therefore, are built on both preconceived notions (stemming from personal or familial experience) and objective open-mindedness (allowing physician intake). We understand how knowledge was formed among the participants and the factors that led to the formation of women's perception of CS, thus the main theme remains on transforming these perceptions into reality.

Decision on Mode of Delivery

Some women asserted their autonomy in the decision-making process, and their husbands' sole concern was the safety of mother and baby. Their words echoed the sentiment of empowerment and safety, highlighting their trust in their decision and the support of their partner.

"The decision to choose the method of delivery is up to me. My husband tells me to do what I feel comfortable about. The important thing is that you and the baby are safe." (Primipara, FG 2)

Similarly, some women showed a sense of independence and self-assurance in expressing their desire for a C-section, committed to their own beliefs and unaffected by external pressures or opinions:

"I want to give birth by CS. I don't feel that those around me are influencing me. I don't listen to their opinions. I told you; I only do what I am convinced of." (Primipara, FG 3)

Other women see the doctor as pivotal in the decision-making process, and they are prepared to change their decisions based on medical advice.

"The doctor is first in line. He knows my condition and that of the fetus. I mean, I like NVD. If he tells me that a CS is better, I will respond." (Primipara, FG 1).

Recommendations for Future Mothers

When asked what recommendations they would give to women about giving birth, most of the participants were aware of the impact of their advice but chose not to express their opinion. They emphasized the importance of informed decision-making guided by medical expertise based on each mother's particular circumstances. They avoided imposing their own experiences on other expectant mothers, honoring their autonomy to make their own decisions.

"I will not express my opinion. She may take my words seriously. I will tell her, "See what suits you and do it. See what the doctor tells you." (Primipara, FG2)

"According to her doctor and his advice. According to her condition and that of her fetus. I won't tell her about my experience." (Para 1, FG3)

Discussion

This study's findings show that cultural, social, and individual factors influence women's perceptions of CS.(12) perceived CS as a necessary medical intervention ensuring the safety of both mother and baby. It is a modern advancement that offers controlled and planned delivery whilst also reducing the risks and anxious wait associated with NVD. In cultures where NVD is traditionally preferred, CS often has negative associations, representing intervention and a deviation from the normal process of childbirth (13). Our findings classed these points under the

perceived risks of CS. Healthcare providers must understand the diverse perspectives of CS to be able to provide tailored support and education to expectant mothers.

Most of our participants perceive CS as a safer and less painful alternative to NVD. A study conducted in Pakistan reported that women chose CS because they feared the pain associated with NVD, and they preferred to schedule the delivery (14). In our study, women self-reported that their obstetricians and their recommendations were a major influence.

Despite some women relying on specific knowledge areas and personal or family experiences to choose their mode of delivery, a power hierarchy exists between them and obstetricians. This often results in medical professionals making the final decision. Women and their husbands frequently expressed trust in the doctors, placing the final decision in their hands. As observed in (15), although pregnant women expressed a preference for NVD, their wishes are often overlooked due to the dominance of CS preference in obstetrics. Moreover, the narrative literature review by Sys et al. (2021) asserted that communication with medical professionals is the key to making an informed decision regarding the mode of delivery. These results can help obstetricians identify and acknowledge their role as crucial members in the decision-making process for CS deliveries within their institutions. They can also form the basis for the development of an intervention that targets women and their husbands during the antenatal period, raising awareness of the benefits of NVD and the risks of CS. Obstetricians and other maternity healthcare providers have a duty to try to change women’s negative perceptions of natural childbirth, so they don’t opt for medically unjustified CS deliveries.

Krychman (2016) opined that vaginal laxity can decrease sexual satisfaction, which can affect a woman's sense of sexual self-esteem and her relationship with her sexual partner. The women in the current study revealed that the fear of vaginal laxity following NVD is a significant

concern for them and their husbands, causing them to opt for CS over NVD. They worry about a loss of vaginal tightness, which they believe could negatively affect sexual satisfaction for both themselves and their husbands. This belief can lead women to reject natural childbirth due to the potential consequences of vaginal delivery, ignoring the higher medical risks and longer recovery associated with CS. It also underscores the mix of psychological and social factors that influence childbirth decisions. A holistic approach is needed to address childbirth's full range of medical, emotional, and relational dimensions. Comprehensive education is called for so that healthcare providers can support women in making informed decisions while reducing the health risks of unnecessary medical procedures.

In conclusion, women hold a complex blend of ideas and perceptions when it comes to modes of birth. Although they have their own perceptions regarding the meaning, benefits, and disadvantages of delivery by Cesarean section, the major influence on their decision-making was ultimately found to be the advice they received from their doctor. A full understanding of the diverse perspectives and preferences of expectant women when it comes to the mode of delivery can potentially inform patient-centered care approaches where women are encouraged to make informed decisions while receiving psychological support and holistic antenatal care.

Acknowledgments

This manuscript is made possible by the support of the American people through the United States Agency for International Development (USAID) in partnership with Ministry of Health. The contents/findings of this manuscript are the sole responsibility of University Research Co., LLC (URC) and do not necessarily reflect the views of USAID or the United States Government.

References

1. Betrán AP, Torloni MR, Zhang J-J, Gülmezoglu A, Aleem H, Althabe F, et al. WHO statement on caesarean section rates. Bjog. 2016;123(5):667.
2. Al-Rawashdeh I, Kharboush I, Al-Kubaisy W. Disparities in cesarean section among women in Jordan: analysis of the 2017-18 Jordan Population and Family Health Survey (JPFHS) data. 2020.
3. Salem A-F. Trends in Caesarean section deliveries in Jordan from 1982 to 2017: retrospective analyses of annual hospital reports. Eastern Mediterranean Health Journal. 2021;27(2).
4. Sandall J, Tribe RM, Avery L, Mola G, Visser GH, Homer CS, et al. Short-term and long-term effects of caesarean section on the health of women and children. The Lancet. 2018;392(10155):1349-57.
5. Malagon-Maldonado G. Qualitative research in health design. HERD: Health Environments Research & Design Journal. 2014;7(4):120-34.
6. Van Manen M. Researching lived experience: Human science for an action sensitive pedagogy: Routledge; 2016.
7. Dworkin SL. Sample size policy for qualitative studies using in-depth interviews. Springer; 2012. p. 1319-20.
8. Fetters MD, Curry LA, Creswell JW. Achieving integration in mixed methods designs—principles and practices. Health services research. 2013;48(6pt2):2134-56.
9. DiCicco-Bloom B, Crabtree BF. The qualitative research interview. Medical education. 2006;40(4):314-21.
10. Sparkes AC, Smith B. Judging the quality of qualitative inquiry: Criteriology and relativism in action. Psychology of sport and exercise. 2009;10(5):491-7.
11. Lincoln YS, Guba EG. Naturalistic inquiry: sage; 1985.
12. Laurita Longo V, Odjidja EN, Beia TK, Neri M, Kielmann K, Gittardi I, et al. “An unnecessary cut?” multilevel health systems analysis of drivers of caesarean sections rates in Italy: a systematic review. BMC pregnancy and childbirth. 2020;20:1-16.
13. Chen I, Opiyo N, Tavender E, Mortazhejri S, Rader T, Petkovic J, et al. Non-clinical interventions for reducing unnecessary caesarean section. Cochrane Database of Systematic Reviews. 2018(9).
14. Hassan MM, Ameeq M, Fatima L, Naz S, Sikandar SM, Kargbo A, et al. Assessing socio-ecological factors on caesarean section and vaginal delivery: an extended perspective among women of South-Punjab, Pakistan. Journal of Psychosomatic Obstetrics & Gynecology. 2023;44(1):2252983.

15. Escobal APdL, Andrade APMd, Matos GCd, Giusti PH, Cecagno S, Prates LA. Relationship between power and knowledge in choosing a cesarean section: women’s perspectives. Revista brasileira de enfermagem. 2021;75:e20201389.

Click here to access/download
Supporting Information
JU IRB.pdf
