# Supplementary material for: An interpretative phenomenological study about maternal perceptions of cesarean birth
Source: PLoS One. 2025 Feb 6;20(2):e0318525. doi: 10.1371/journal.pone.0318525 (PMC11801696; doi:10.1371/journal.pone.0318525)
Supplement: Attachment — Submitted filename: Replay to reviewers comments.doc [file pone.0318525.s003.doc]

Replay to reviewers comments

| Comments | Replay | Page number |
| --- | --- | --- |
| Methods – line 97 indicates that only focus group discussion were done | "Yes, only seven focus groups were conducted. All instances of 'in-depth interview' in the text were changed to 'focus group discussions (FGDs).'" | Page 2+ 5+ 6 |
| Results: I think that it is better if the theme headings come from the content of the interviews – I am not sure that women would be using the words: cognitive structures and transforming perceptions into practice | Thank you for highlighting this aspect. In interpretive phenomenological analysis (IPA), the researcher’s role includes making sense of participants' experiences through their interpretations, which often involve conceptualizing themes beyond participants' language. The themes reflect the researcher's analytical depth and insight into the phenomenon rather than staying strictly at the participants' level of language and knowledge. This study's analysis was based on our interpretations and intended to provide deeper insights into the participants’ experiences rather than mirroring their exact language. The thematic headings aim to capture the essence of the experiences from participants' narratives. Your feedback is highly appreciated and respected. |  |
| Introduction  An overview of the health care services in Jordan would be helpful. How much say do women have in whether or not they have a CS? If they would prefer a CS is this taken into consideration or are most CS done for medical indications? Who provides antenatal care? What is covered during antenatal care? | Thank you for these valuable suggestions. We agree that including an overview of the healthcare services in Jordan, specifically regarding maternal and childbirth care, would enrich the context of the study. We added a section detailing the healthcare structure, accessibility, and the provision of maternal services. We will also address the degree of autonomy women have in deciding to undergo a cesarean section. Thank you again for your helpful comments. Incorporating the context of maternal health care services will strengthen the contextual background and enhance the study’s relevance and clarity. The following paragraphs are added to the manuscript:  In Jordan, maternity care encompasses both public and private healthcare services, supporting women through pregnancy, childbirth, and the postpartum period. While many women utilize public health facilities due to their accessibility and affordability, a significant number choose private healthcare for its perceived higher quality and personalized care, despite the associated higher costs. Public hospitals often remain the primary choice for complex pregnancies or labor due to their comprehensive services **(Khader et al., 2018; Alyahya et al., 2019).**  Maternity care services are provided by obstetricians, nurses, and midwives across both healthcare sectors. Notably, 96% of women prefer to receive antenatal care from a doctor, with only 3% selecting for midwives or nurses, indicating a strong preference for physician-led services. Antenatal visits typically include essential check-ups, screenings, nutritional advice, and health education. The experience can vary based on the chosen healthcare setting: private facilities often provide longer consultations, which many women find more reassuring, while public services are favored for their affordability and availability **(Alyahya et al., 2019).**  Women’s perceptions and decisions about CS are shaped by personal beliefs, cultural influences, and healthcare setting. Some women view CS as a necessary medical intervention to ensure the safety of both mother and baby, perceiving it as a modern advancement that offers controlled and planned delivery while potentially reducing the risks associated with normal vaginal delivery (NVD). However, this perception is often influenced by factors such as trust in doctors, advice from healthcare providers, and familial or cultural expectations, particularly in private hospitals where personalized care is emphasized **(Fram et al., 2023).** | Page 3+ 4 |
| Why were primips chosen? They may have perceptions, but they don’t have any experience of giving birth either vaginally or via CS | Thank you for your valuable comment. One of the aims of the study was to understand women's perspectives on the cesarean section. To achieve this, we included both primiparous women and those who had given birth once (para 1). Primiparous women were specifically included because their perceptions are influenced by societal, cultural, and healthcare-related factors, which shape their opinions and expectations about cesarean birth, even without personal birthing experience. Interviews conducted during pregnancy provided an opportunity to explore their perspectives and decision-making processes before they experienced childbirth.  Additionally, the inclusion of women who had given birth once allowed us to examine how these initial perceptions may change after their first birth experience, regardless of the mode of delivery. This approach enabled a more comprehensive understanding of maternal perspectives on cesarean sections, both before and after giving birth. We believe this diversity in the sample enriched the study by capturing the full picture of experiences and expectations related to cesarean delivery. |  |
| I am also not sure how women’s perspectives – particularly if they are wanting a CS for non-medical reasons, promote optimal maternal and neonatal outcomes (line 71 – 72). I do understand that there is a need for mutual dialogue and a shared understanding of the reason for CS. | Thank you for your insightful comment. We appreciate your comment regarding how women's perspectives, particularly in cases where they desire a cesarean section for non-medical reasons, align with promoting optimal maternal and neonatal outcomes.  Our intent in this section was to highlight the importance of understanding maternal perspectives and encouraging a discussion between women and healthcare providers. We recognize that decisions for cesarean sections driven by non-medical reasons may not always align with optimal clinical outcomes. However, we believe that engaging in mutual discussion and establishing a shared understanding of the reasons for cesarean delivery is essential in ensuring that women are well-informed about potential risks and benefits. This collaborative approach supports decision-making that prioritizes both maternal preferences and evidence-based practices for maternal and neonatal health.  To clarify, in our recommendations, we mentioned the importance of informed decision-making and the role of healthcare providers in guiding women toward choices that support optimal outcomes while respecting their preferences. Thank you for bringing this to our attention. |  |
| The purpose of the study was to provide insight for clinical practice, policy development and future practice (line 81 – 82). In the conclusions (line 390 – 396) I cannot find reference to how the finding contribute to policy development | We appreciate your feedback on ensuring that the conclusions adequately address how the findings contribute to policy development. We agree that linking the findings to policy development is essential to fulfill the stated purpose of the study.  To address this, we will revise the conclusion section to clearly outline how the study findings can inform policy development in maternity care. Specifically, we will highlight the importance of incorporating women's perspectives into policymaking to create patient-centered approaches, improve informed consent processes, and address non-medical cesarean section requests through education and counseling policies.  This is the section we added to the conclusion:  Furthermore, the findings of this study highlight the importance of integrating women's perspectives into policy development to enhance patient-centered maternity care. Policies should prioritize shared decision-making between women and healthcare providers, emphasizing the importance of informed consent and providing clear, evidence-based information about cesarean delivery and its implications. Additionally, the study underscores the need for policies that support education and counseling programs to address non-medical requests for cesarean sections, ensuring that maternal and neonatal health outcomes are optimized. These findings can guide the development of maternity care policies in Jordan and similar contexts, promoting better alignment between women's preferences and evidence-based clinical practices | Page 22 |
| Setting and participants  Lines 57 – 60 suggest that high rates of CS are found in University hospitals (40,4%) Private hospitals (39,1%), Military hospitals (36,1%) and government hospitals (27,4%) but participants were chosen from 2 public hospitals and 2 health centres (do they even do CS at health centres?). Why were these centres chosen and not those with high rates of CS? | Thank you for your comment. We appreciate the opportunity to clarify the rationale behind our selection of settings and participants for this study.  While high rates of cesarean sections were noted in university, private, and military hospitals, we aimed to gather a representative sample of perspectives from women across various healthcare settings in Jordan. To achieve this, we included participants from one military hospital and one teaching hospital, both of which perform cesarean sections, and complemented this with participants from two health centers. Although health centers do not provide cesarean sections, they offer postpartum care and newborn vaccinations, making them an important point of contact for mothers who have recently given birth. This approach allowed us to reach women who delivered in various types of hospitals, including private and government hospitals, thereby capturing a broader range of views and experiences regarding cesarean sections.  We believe this sampling technique (maximum variation sampling) enabled us to gather diverse insights into maternal perceptions of cesarean birth across Jordan, reflecting the different healthcare contexts where women receive maternity care. |  |
| Line 92 – 93 - 3 +4 = 7 Focus groups while the abstract suggests that in depth semi structured interviews were also done (line 39 -40). Please clarify | Thank you for pointing out this inconsistency. We appreciate your careful review and feedback on this matter.  To clarify, we conducted 7 focus group interviews, all of which were guided by a semi-structured format. The phrasing "in-depth semi-structured" was mistakenly included in the manuscript, which may have led to confusion. To address this, we have deleted all references to "in-depth semi-structured" from the manuscript and ensured that the description of the methodology is consistent throughout.  We hope this revision resolves the issue and provides clarity regarding the methodology used in this study. Thank you for bringing this to our attention. | Page 2+ 5+ 6 |
| Line 95 talks about maximum variation – It is not clear what factors were considered in selecting women with maximum variation. Age range, and employment are reported in the results but not education or family income | Thank you for your valuable comment. We appreciate the opportunity to clarify the factors considered in achieving maximum variation in participant selection.  In selecting participants, we aimed to capture diverse experiences and perspectives regarding cesarean birth. The primary factors considered for maximum variation included age, parity (primipara vs. para 1), employment status (employed vs. housewives), and the type of antenatal care (public, private, or mixed). While we did not specifically include education level or family income, the diversity in the settings from which participants were recruited from public hospitals, military hospitals, teaching hospitals, private hospitals, and health centers—likely contributed to variability in socioeconomic backgrounds. |  |
| Line 97 – it is not clear how theme saturation influences the sample size. Where there interviews, then analysis and theme identification, followed by more interviews, analysis and theme identification? | Thank you for your comment, regarding the concept of 'theme saturation' and its influence on sample size. We appreciate the opportunity to clarify our methodology and address the terminology used.  We acknowledge that the term 'theme saturation' may not be standard terminology in qualitative research. The more widely accepted term is 'data saturation,' which refers to the point in data collection when no new information or themes emerge, indicating that the data is sufficiently rich and comprehensive. We will revise the manuscript to replace 'theme saturation' with 'data saturation' to align with established qualitative research terminology.  Thank you for bringing this to our attention, and we will make the necessary revisions to enhance the clarity and accuracy of our manuscript. | Page 6 |
| Line 100 – please clarify if in depth interview were done. It would appear that 7 focus groups X 5 – 7 members = the 41 participants | Thank you for pointing out this inconsistency. We appreciate your careful review and feedback on this matter.  To clarify, we conducted 7 focus group interviews, all of which were guided by a semi-structured format. The phrasing "in-depth semi-structured" was mistakenly included in the manuscript, which may have led to confusion. To address this, we have deleted all references to "in-depth semi-structured" from the manuscript and ensured that the description of the methodology is consistent throughout.  We hope this revision resolves the issue and provides clarity regarding the methodology used in this study. Thank you for bringing this to our attention. | Page 2+ 5+ 6 |
| Line 102 – it is not clear how the questions were developed and what questions were asked. These should be included as an appendix | Thank you for your comment. The research team developed a semi-structured interview guide to explore maternal perceptions of cesarean birth. This guide was informed by a comprehensive review of existing literature on maternal health and cesarean delivery, as well as consultations with experts in obstetrics and qualitative research methodologies. To provide a comprehensive understanding of our methodology, we will include the complete interview guide as an appendix in the revised manuscript. This addition will offer readers detailed insight into the specific questions posed during the interviews. We hope that this clarification addresses your concerns, and we appreciate your constructive feedback, which has contributed to improving the clarity and rigor of our study. | Page 6 |
| Line 105 – when did the focus group discussions take place? | The dates of the interviews has been added: “Participant recruitment commenced after obtaining IRB approval and took place from February 1 to March 30, 2024.” | Page 6 |
| Line 107 – it is not clear how the selection of participants was done. What was the process of selecting participants | Thank you for your insightful comment regarding the participant selection process. To ensure a diverse and representative sample, we employed a purposive sampling strategy, focusing on maximum variation to capture a wide range of maternal perspectives on cesarean birth. Women who met the inclusion criteria were invited to participate in the study. |  |
| Line 130 – who were the WhatsApp sent to? | Thank you for your insightful question regarding the recipients of the WhatsApp messages mentioned in line 130. We appreciate the opportunity to clarify this aspect of our methodology.  During the study, several participants from the primipara focus groups 2 and 3, as well as from the para 1 focus groups 2 and 4, expressed interest in reviewing the final list of themes derived from the interviews. To facilitate this, we utilized WhatsApp to share the thematic summaries with these participants, enabling them to provide feedback and ensure the accuracy of the findings with their experiences. We will revise the manuscript to include this clarification.  Thank you for bringing this to our attention. This is the revised text:  The first author discussed the emerging themes and their interpretations with the research team, taking their feedback into account. During the study, several participants from the primipara focus groups 2 and 3 and the para 1 focus groups 2 and 4 expressed interest in reviewing the final list of themes derived from the interviews. To facilitate this, the thematic summaries were shared via WhatsApp with these participants, enabling them to provide feedback and ensure the accuracy of the findings with their experiences. This process ensured that participants' opinions, experiences, and needs were accurately represented. | Page 7 |
| Results  It would be important to know how many of the para 1 women had delivered by CS | Thank you for your valuable comment regarding the importance of understanding how many para 1 women are delivered by CS. Our sample comprised 17 primiparas and 24 para 1 participants, of whom 15 had delivered by cesarean section. This breakdown enriches the context of our findings, particularly regarding the diverse experiences and perceptions surrounding cesarean delivery. We have now incorporated this detail into the revised manuscript to ensure clarity and comprehensiveness. | Page 8 |
| Line 142 – this is a limitation of qualitative research and should be placed at the end of the manuscript | Thank you for your comment regarding the content on line 142. We acknowledge that the discussed point aligns with limitations typically observed in qualitative research, such as the contextual specificity of participants' perceptions and the non-representative nature of qualitative samples. In response to your suggestion, we will relocate this content to the limitations section of the manuscript to better align with standard reporting practices. | Page 22 |
| Line 146 – this suggests that women are the primary decision maker in whether or not a CS is performed. Is this true? | Thank you for your comment. We appreciate your insight regarding the decision-making process for cesarean sections. To address this concern, we have clarified the statement to better reflect the shared nature of the decision-making process. The revised text now is:  "We found that women generally followed an individualized decision-making process regarding CS, shaped by their knowledge, personal context, and several influencing factors while placing significant trust in their doctors' recommendations and engaging in a collaborative decision-making process.”  This adjustment highlights that while women form perceptions and preferences regarding the mode of delivery, they typically place significant trust in their doctors and their recommendations when faced with the actual situation. Ultimately, the decision is often a collaborative one, shaped by medical advice and women’s circumstances.  We believe this revised phrasing better aligns with the findings of the study and accurately represents the shared decision-making dynamic. | Page 21+ 22 |
| Line 168 – the women is talking about both CS and NVD – she is Para 1 and can only have experienced either NVD or CS | Thank you for your comment. While it is correct that a para 1 woman can only have experienced either NVD or CS, the statement reflects her perception of both modes of delivery. Perception, as we understand it, is not solely shaped by direct personal experience; it also encompasses information from other sources, such as conversations with family or friends, cultural narratives, and information she may have read or heard from healthcare providers or media.  This quote illustrates how perceptions of childbirth are complex and multidimensional, often combining direct experiences with external influences. |  |
| Line 170 suggests that the choice for a CS is made by the women – this true? | Thank you for your comment regarding Line 170. The statement in the manuscript reflects the women's preferences for the mode of delivery when they were asked about it during the interview. It is important to clarify that the participants were expressing their perspectives and preferences rather than recounting an actual decision made during childbirth. Specifically, some primipara participants expressed a preference for cesarean section (CS) based on their perceptions that it would involve less suffering and pain before seeing the baby, despite their understanding of the potential pain associated with recovery after CS. |  |
| Line 173 – I prefer a CS – this is a primip speaking | Thank you for your comment regarding Line 173. Yes, the participant quoted was Primipara. The study specifically aimed to explore the perceptions of both primipara and para 1 women regarding their preferred mode of delivery. We intentionally included primipara and para 1 women in the study because they still have the opportunity to choose their delivery mode without being medically required to choose a cesarean section (CS), as is often the case after two CS deliveries. This allowed us to capture their preferences and the underlying justifications for those preferences before such decisions are influenced by clinical necessity. |  |
| Line 189 – this is a perception of the care provided during labour – from a primip who has no experience | Thank you for your comment regarding Line 189. The quote provided reflects the perspective of a primiparous woman (primipara) who has not personally experienced labor but has developed a perception of the care provided during labor based on various sources. In this study, our aim is to explore women’s perceptions of their preferred mode of delivery, regardless of their personal obstetric experience. A woman’s perception is shaped not only by her own experiences (if she has any) but also by the narratives she encounters through her social network, including stories from female family members, friends, relatives, and neighbors. Additionally, perceptions may be influenced by what women read and by the advice or information they receive from maternity care providers. This context explains why a primiparous woman, despite lacking direct labor experience, can still articulate a detailed perception of labor care that informs her preference for cesarean section. |  |
| Line 213 – the quote is accredited to a primip but the actual quote is from a para 1 (line 216) | Thank you for pointing out the discrepancy in the quote in Line 213. You are correct that the quote is from a para 1 and not a primipara, as wrongly stated. This was an unintentional mistake, and we will revise the text to correctly attribute as follows: “One para 1 added that she was afraid of infection in the CS wound and was concerned about how to care for it.” We appreciate your careful review and attention to detail in identifying this mistake. | Page 12 |
| Line 324 – who makes the decision about CS birth? Under what circumstances can the women ‘ assert their autonomy’ (line 324) in this process? | Thank you for your comment regarding Line 324. In our paper, we discussed that some women assured their autonomy in the decision-making process regarding the mode of delivery, particularly in relation to their husbands. As we mentioned, this was not the case for all women in the study. Some women clearly stated that they were the primary decision-makers, and their husbands’ sole concern was ensuring the safety of the mother and baby. This reflects a dynamic where husbands provided support rather than imposing a preference, leaving the decision to the woman and giving significant weight to the doctor’s advice. However, we acknowledge that this scenario was not universal across all participants in the study, as decision-making processes varied depending on individual circumstances and family dynamics. |  |
| Discussion  Line 352 – it is not clear how a womens perception of a CS ensures the safety of both mother and child. Ideally there should be mutual sharing of information between Doctor and patient so that there is a common understanding of the reasons for a CS which should be medical indications rather than factors of convenience. | Thank you for your comment regarding Line 352. We appreciate your argument about the importance of ensuring that decisions for a cesarean section (CS) are based on medical indications rather than convenience or misperceptions. To address this, we will revise the manuscript to clarify how women’s perceptions of CS relate to safety and to emphasize the importance of mutual sharing of information between doctors and patients. We will highlight that healthcare providers play a critical role in providing evidence-based guidance to ensure that women’s perceptions and decisions are informed by accurate medical indications.  Additionally, we will elaborate on the point that women’s perceptions of CS may be influenced by cultural, social, and individual factors, and not necessarily by medical necessity. We will include the importance of doctor-woman communication in fostering a shared understanding of the reasons for CS, ensuring that women are empowered to make informed decisions based on their unique needs and circumstances.  We will revise the manuscript as follows to reflect these clarifications:  This study’s findings show that cultural, social, and individual factors influence women's perceptions of CS. (12) Some women perceived CS as a necessary medical intervention to ensure the safety of both mother and baby, viewing it as a modern advancement that offers controlled and planned delivery while potentially reducing risks associated with NVD. However, this perception is not always grounded in medical necessity but may stem from fear, misinformation, or cultural beliefs. It is critical for healthcare providers to engage in mutual, open communication with expectant mothers, ensuring that decisions for CS are based on accurate medical indications rather than convenience or societal perceptions. In cultures where NVD is traditionally preferred, CS often carries negative associations, representing intervention and a deviation from the normal process of childbirth (13). Healthcare providers must acknowledge and address these diverse perspectives to provide tailored support, education, and informed decision-making to expectant mothers. | Page 19 |
| The layout of the discussion does not follow the same format as the results are presented:  Knowledge – meaning of CS, perceived benefits, perceived concerns, previous experiences  Those influencing the perception: trust in the doctor, influence of husband and gender equity, role of culture, maternal health conditions  Transforming perceptions into action: decisions on mode of delivery, recommendations for future mothers  The discussion is not structured in the same order as the results are presented | Thank you for your comment regarding the layout of the discussion section. While it is not a strict requirement in qualitative research for the discussion to follow the exact same order as the results, some qualitative research guidelines emphasize that aligning the structure of the discussion with the results can enhance clarity and readability. However, it is also common for qualitative discussions to take a more thematic or integrative approach, particularly when synthesizing findings to address the study’s overarching objectives or to frame recommendations. In this study, we chose to structure the discussion based on thematic priorities rather than the exact order of results to allow for a more solid analysis of the broader implications of the findings. |  |
| Line 368 is not complete | Thank you for pointing out that Line 368 is incomplete. Upon review, we recognize that the sentence lacks proper context or elaboration to fully convey its intended meaning. To address this, we will revise the sentence to provide a more complete explanation, ensuring that it accurately reflects the findings of the referenced study and aligns with the broader discussion of our research.  Revised Text for Line 412-416:  “As observed in (15), although pregnant women expressed a preference for NVD, their wishes are often overlooked due to the dominance of CS preference in obstetrics, which can stem from institutional protocols, practitioner convenience, or liability concerns. This underscores the need for healthcare providers to prioritize patient-centered care, ensuring that women’s preferences for NVD are respected and supported whenever medically appropriate.” | Page 20 |
|  |  |  |
