# Supplementary material for: An interpretative phenomenological study about maternal perceptions of cesarean birth
Source: PLoS One. 2025 Feb 6;20(2):e0318525. doi: 10.1371/journal.pone.0318525 (PMC11801696; doi:10.1371/journal.pone.0318525)
Supplement: Attachment — Submitted filename: Feedback on submission.docx [file pone.0318525.s001.docx]

**An Interpretative Phenomenological Study about Maternal Perceptions of Cesarean**

**Birth**

Feedback on submission

Technically sound:

Data availability: Ethics board approval is attached. However the transcripts of the interviews are not available for review

Language: There is need for some English editing

**Abstract**

Background: Understanding these factors is essential for informing healthcare practices and promoting optimal maternal and neonatal health outcomes.

Methods – line 97 indicates that only focus group discussion were done

Results: I think that it is better if the theme headings come from the content of the interviews – I am not sure that women would be using the words: cognitive structures and transforming perceptions into practice

Introduction

An overview of the health care services in Jordan would be helpful. How much say do women have in whether or not they have a CS? If they would prefer a CS is this taken into consideration or are most CS done for medical indications? Who provides antenatal care? What is covered during antenatal care?

Why were primips chosen? They may have perceptions but they don’t have any experience of giving birth either vaginally or via CS

I am also not sure how women’s perspectives – particularly if they are wanting a CS for non-medical reasons, promote optimal maternal and neonatal outcomes (line 71 – 72). I do understand that there is a need for mutual dialogue and a shared understanding of the reason for CS.

The purpose of the study was to provide insight for clinical practice, policy development and future practice (line 81 – 82). In the conclusions (line 390 – 396) I cannot find reference to how the finding contribute to policy development

Setting and participants

Lines 57 – 60 suggest that high rates of CS are found in University hospitals (40,4%) Private hospitals (39,1%), Military hospitals (36,1%) and government hospitals (27,4%) but participants were chosen from 2 public hospitals and 2 health centres (do they even do CS at health centres?). Why were these centres chosen and not those with high rates of CS?

Line 92 – 93 - 3 +4 = 7 Focus groups while the abstract suggests that in depth semi structured interviews were also done (line 39 -40). Please clarify

Line 95 talks about maximum variation – It is not clear what factors were considered in selecting women with maximum variation. Age range, and employment are reported in the results but not education or family income

Line 97 – it is not clear how theme saturation influences the sample size. Where there interviews, then analysis and theme identification, followed by more interviews, analysis and theme identification?

Line 100 – please clarify if in depth interview were done. It would appear that 7 focus groups X 5 – 7 members = the 41 participants

Line 102 – it is not clear how the questions were developed and what questions were asked. These should be included as an appendix

Line 105 – when did the focus group discussions take place?

Line 107 – it is not clear how selection of participants was done. What was the process of selecting participants

Line 130 – who were the whattsapps sent to?

**Results**

Womens perceptions do need to be taken into consideration and there needs to be dialogue to ensure that women understand the reasons for the decisions regarding CS delivery.

It would be important to know how many of the para 1 women had delivered by CS

Line 142 – this is a limitation of qualitative research and should be placed at the end of the manuscript

Line 146 – this suggests that women are the primary decision maker in whether or not a CS is performed. Is this true?

Line 168 – the women is talking about both CS and NVD – she is Para 1 and can only have experienced either NVD or CS

Line 170 suggests that the choice for a CS is made by the women – this true?

Line 173 – I prefer a CS – this is a primip speaking

Line 189 – this is a perception of the care provided during labour – from a primip who has no experience

Line 213 – the quote is accredited to a primip but the actual quote is from a para 1 (line 216)

Line 270 – it is not clear why the doctor thought that she would have complications

Line 324 – who makes the decision about CS birth? Under what circumstances can the women ‘ assert their autonomy’ (line 324) in this process?

**Discussion**

Line 352 – it is not clear how a womens perception of a CS ensures the safety of both mother and child. Ideally there should be mutual sharing of information between Doctor and patient so that there is a common understanding of the reasons for a CS which should be medical indications rather than factors of convenience.

The layout of the discussion does not follow the same format as the results are presented:

**Knowledge** – meaning of CS, perceived benefits, perceived concerns, previous experiences

**Those influencing the perception**: trust in the doctor, influence of husband and gender equity, role of culture, maternal health conditions

**Transforming perceptions into action**: decisions on mode of delivery, recommendations for future mothers

The discussion is not structured in the same order as the results are presented

Line 368 is not complete

Line 369 – I am not sure that this is true (it might be in some contexts)
